# Magnetic-Field-Assisted Electric-Field-Induced Domain Switching of a Magnetic Single Domain in a Multiferroic/Magnetoelectric Ni Nanochevron/[Pb(Mg_1/3_Nb_2/3_)O_3_]_0.68_–[PbTiO_3_]_0.32_ (PMN–PT) Layered Structure

**DOI:** 10.3390/mi15010036

**Published:** 2023-12-23

**Authors:** Chih-Cheng Cheng, Yu-Jen Chen, Shin-Hung Lin, Hsin-Min Wang, Guang-Ping Lin, Tien-Kan Chung

**Affiliations:** 1 Department of Mechanical Engineering, National Yang Ming Chiao Tung University, Hsinchu 30010, Taiwan; jjcheng.en03@nycu.edu.tw (C.-C.C.); a311611117.en11@nycu.edu.tw (G.-P.L.); 2 Electronic and Optoelectronic System Research Laboratories, Industrial Technology Research Institute, Hsinchu 310401, Taiwan; 3 International College of Semiconductor Technology, National Yang Ming Chiao Tung University, Hsinchu 30010, Taiwan; 4 Institute of Advanced Semiconductor, National Yang Ming Chiao Tung University, Hsinchu 30010, Taiwan

**Keywords:** magnetoelectric, multiferroic, nano Ni chevrons, piezoelectric PMN–PT, electric-field control, magnetic single domain, domain switching, domain transformation, nanoelectromagnet

## Abstract

We report the magnetic-field-assisted electric-field-controlled domain switching of a magnetic single domain in a multiferroic/magnetoelectric Ni nanochevrons/[Pb(Mg_1/3_Nb_2/3_)O_3_]_0.68_–[PbTiO_3_]_0.32_ (PMN–PT) layered structure. Initially, a magnetic field was applied in the transverse direction across single-domain Ni nanochevrons to transform each of them into a two-domain state. Subsequently, an electric field was applied to the layered structure, exerting the converse magnetoelectric effect to transform/release the two-domain Ni nanochevrons into one of two possible single-domain states. Finally, the experimental results showed that approximately 50% of the single-domain Ni nanochevrons were switched permanently after applying our approach (i.e., the magnetization direction was permanently rotated by 180 degrees). These results mark important advancements for future nanoelectromagnetic systems.

## 1. Introduction

The development of nanoscale electromagnetic systems (or the ability to electrically generate a magnetic field at the nanoscale, similar to the function of a nanoelectromagnet) is important and would enable a variety of novel applications such as magnetically/electromagnetically actuated cell manipulation [1,2], nano/microantennas for emitting and receiving magnetic/electromagnetic waves [3], and other magnetically/electromagnetically driven biomedical microelectromechanical systems/nanoelectromechanical systems (MEMS/NEMS) [4,5,6,7,8,9]. However, developing nanoscale electromagnetically actuated or electrically generated magnetic fields is still challenging for micro/nanosystems and MEMS/NEMS researchers. Therefore, these researchers are pursuing new mechanisms/structures to enable the electromagnetic actuation or electrical generation of magnetic fields at the nanoscale.

Recently, analytical models and experimental approaches for multiferroic/magnetoelectric materials/structures have been studied in the context of memory devices [10,11,12,13,14,15,16,17,18,19]. These approaches use the converse magnetoelectric effect found in multiferroic and magnetoelectric materials/structures [20,21,22,23,24,25,26,27]. When an electric field is applied to these materials/structures, mechanical strain is generated due to the piezoelectric effect. Furthermore, due to magnetostriction, this strain transforms the magnetic domains of the material/structure, which in turn changes its magnetization and magnetic field [3,4,5,6,7,8,9,10,11,12,13,14]. Most researchers believe that the coupling of electric fields, strain, and magnetic domains/magnetization (as well as magnetic fields) could be used to electrically control magnetic domains/magnetization (and electrically generate/control magnetic fields) at the nanoscale. However, few researchers have successfully demonstrated this (i.e., electrically controlling the magnetic domains in multiferroic/magnetoelectric materials at the nanoscale) [4,5,6,7,8,18,19,20,21].

Zavaliche et al. proposed a self-assembled multiferroic/magnetoelectric particulate composite structure that demonstrates the magnetic-field-assisted electric-field-controlled domain switching of a magnetic single domain [15,23]. A magnetic bias field was applied to change the easy axis of each domain in a single-domain cluster, and an electric field was subsequently applied to induce further domain transformation through the converse magnetoelectric effect. By coupling the magnetic-field-assisted easy-axis change with electric-field-induced domain transformation, half of the domains were eventually “switched” (i.e., the magnetization directions of the domains were rotated 180 degrees). However, due to the configuration of the particulate composite, interaction effects between the domains could not be eliminated; thus, these results were qualitatively similar to the control of multidomain rather than single-domain structures. The constraints on a single-domain structure are much more stringent due to the near-field boundary conditions imposed by the local geometry and magnetic flux conditions (contributed by neighboring domains). Nevertheless, this approach switched approximately 50% of the single-domain clusters (i.e., rotated the magnetization direction by 180 degrees) in the particulate composite.

Ryu et al. [28] demonstrated that the converse magnetoelectric effect in layered structures is generally much stronger than that in particulate composite structures. Recently, several researchers have demonstrated electric-field-induced single-domain transformation in multiferroic/magnetoelectric layered structures [4,5,6,18,22]. However, the single-domain state could be partially transformed (i.e., the domain pattern could be partially changed) but not switched (the magnetization of the domains could not be rotated by 180 degrees). More recently, our laboratory used the above-mentioned magnetic-field-assisted method (i.e., assisting an electric field) to control the magnetization of a multidomain (i.e., stripe-domain) cluster in a multiferroic/layered structure (i.e., a magnetic micro-stripe thin film on a piezoelectric substrate) [13]. Although our laboratory proved that the magnetic-field-assisted electric field method could help rotate the magnetization of the layered structure, our laboratory only demonstrated the control of a multidomain/stripe-domain cluster (partially transforming the multiple magnetic stripe domains, similar to previous work mentioned above). Therefore, the magnetic-field-assisted electrical switching of a single domain in a layered structure remains to be demonstrated.

Recently, Taniyama et al. experimentally demonstrated an interesting magnetic domain transformation in a geometrically patterned zigzag-shaped magnetic microwire [29]. By applying a magnetic field (without an electric field) to the microwire along the transverse direction, the corner of the microwire was transformed to a two-domain (multidomain) state [29,30,31,32]. This two-domain state was relatively unstable; thus, by applying a magnetic field to the microwire along the axial direction, it could be transformed (or “released”) back to the original domain state.

By redesigning the above-mentioned magnetic zigzag microwire, we propose a novel converse magnetoelectric-effect-based multiferroic/magnetoelectric layered structure to demonstrate single-domain switching. We used one segment of the magnetic zigzag microwire with only one convex corner to form a new single-domain nanochevron, which could be transformed to the two-domain state by applying a directional magnetic field. Consequently, we fabricated this nanochevron on a piezoelectric [Pb(Mg_1/3_Nb_2/3_)O_3_]_0.68_–[PbTiO_3_]_0.32_ (PMN–PT) substrate to form the converse magnetoelectric-effect-based multiferroic/magnetoelectric layered structure. Finally, using the above-mentioned approach of sequentially applying magnetic and electric fields to this layered structure, we demonstrated a novel method for magnetic-field-assisted electric-field-controlled magnetic single-domain switching in a multiferroic/magnetoelectric layered structure.

We designed and fabricated a converse magnetoelectric-effect-based multiferroic/magnetoelectric Ni nanochevron/PMN–PT layered structure to demonstrate the magnetic-field-assisted electrical switching of a magnetic single domain. Using electron-beam lithography for nanoscale fabrication, we precisely constructed the nanochevrons and consequently switched individual domains in each of them, solving the magnetic constraint problems caused by neighboring domains in the single-domain clusters in previous studies. In brief, our approach achieved 180° magnetic domain switching/rotation, with the structure subsequently remaining in a relatively stable magnetic domain state. This technique could be used for developing nanoscale electromagnetic/magnetic actuators and data storage/memory devices.

## 2. Design

**Figure 1:**
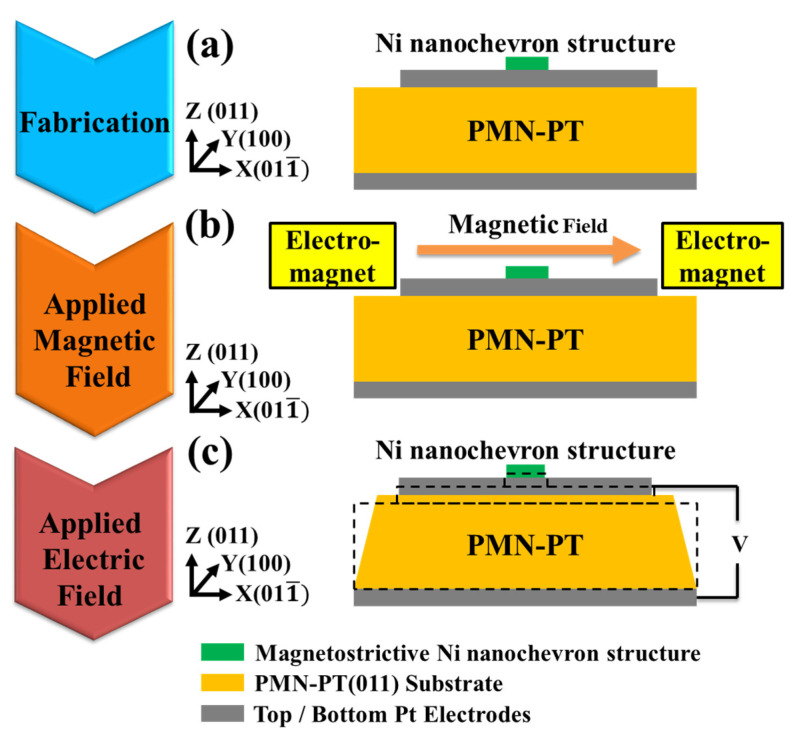
Illustration of our multiferroic/magnetoelectric Ni nanochevron/[Pb(Mg_1/3_Nb_2/3_)O_3_]_0.68_–[PbTiO_3_]_0.32_ (PMN–PT) layered structure and corresponding procedure/principles of our approach (**a**) Fabricating the sample. (**b**) Applying and then removing an axial magnetic field of 3000 Oe to magnetize the domain state in the Ni nanochevron. (**c**) An electric field is applied to the PMN–PT to transform (or “release”) the magnetic domain state of the Ni nanochevron.

The multiferroic/magnetoelectric layered structure, illustrated in Figure 1a, consisted of a single magnetostrictive Ni nanochevron, Pt top and bottom electrodes, and a piezoelectric substrate. The piezoelectric substrate was single-crystal (011) PMN–PT, and the crystallization orientation was the same as that in our previous work [13]. The geometry of the Ni nanochevron was chosen to represent a single-magnetic-domain structure with the preferred magnetization in the direction of the long axis [29]. The magnetic-field-assisted electric-field-induced switching approach proposed for the single-domain Ni nanochevron is shown in Figure 1b,c.

**Figure 2:**
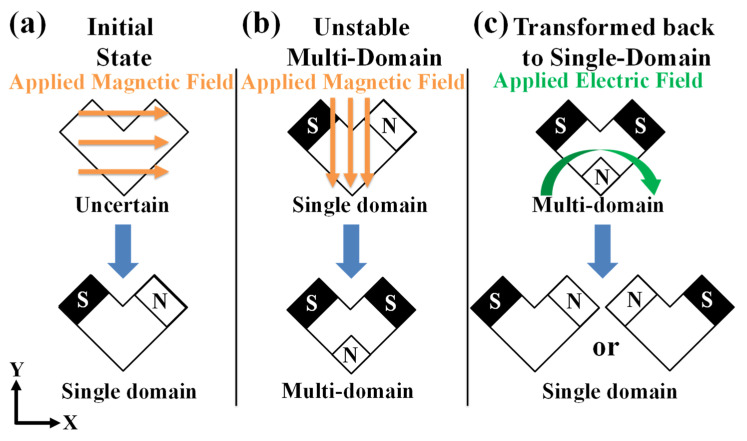
The principles of our approach to magnetic domain transformation in the Ni nanochevrons: (**a**) A positive x-axial magnetic field is applied to initialize/magnetize the magnetic domains in the Ni nanochevron, achieving a single-domain state. (**b**) A negative y-axial magnetic field is applied to transform the initialized/magnetized single-domain state to a multidomain state (i.e., a two-domain state). (**c**) After removing the magnetic field, an electric field is applied to the PMN–PT layer, producing a converse magnetic effect to transform (or “release”) the multidomain state of the Ni nanochevron back to its single-domain state. This single-domain state will be one of two possible magnetization directions.

Furthermore, expanding upon Figure 1, additional details of our domain switching approach are described below (also shown in Figure 2). In the initial state (magnetized), as shown in Figure 2a, the magnetization of the single-domain state followed the long axis (x-axis in the figure) of the Ni nanochevron. After checking the initial state, a magnetic field was applied to the Ni nanochevron to transform the original stable single-domain state into an unstable/metastable two-domain state (a typical multidomain state), as shown in Figure 2b. After this, an electric field was applied to the PMN–PT substrate to produce a modified converse magnetoelectric effect, which transformed the two-domain Ni nanochevron, as shown in Figure 2c. To generate the modified converse magnetoelectric effect, an electric field was applied to the PMN–PT substrate to produce a strain in this layer due to the piezoelectric effect. Through mechanical coupling, the strain was transmitted from the PMN–PT layer to the Ni nanochevron. Finally, due to magnetostriction, the strain influenced and then transformed the magnetic domain pattern of the Ni nanochevron. Our approach of using strain to influence and then naturally transform the domain pattern differs slightly from the conventional application of the converse magnetoelectric effect, which uses strain to control and then precisely transform the domain pattern. We only used strain to influence (stimulate) the change from an unstable state to a naturally stable state. Thus, we did not need to consider the complete coupling of the electric field, strain, and magnetic domains pertinent to the conventional converse magnetoelectric effect. That is, we did not need to determine the direction of the strain produced by the piezoelectric layer and how it would be transmitted to the magnetostrictive layer to control the direction of the domain transformation. Instead, we only needed to use the strain to influence (stimulate) the domain pattern’s natural transformation, which was much easier than the above-mentioned conventional approaches.

After removing the electric field, the two-domain state was further transformed (or “released”) to a final single-domain state, as shown in Figure 2c. This process is similar to using a magnetic field to raise the magnetic domains’ energy level to the unstable peak state (multidomain state) and then applying an electric field to release the unstable peak state to the ground stable state (single-domain state). When comparing the final and initial states (Figure 2a,c), the magnetization of approximately 50% of the single-domain Ni nanochevrons should be switched. Here, switching is defined as the rotation of the single-domain Ni nanochevron’s magnetization direction to its opposite direction, i.e., rotation by 180 degrees. Moreover, our approach can maintain the switched magnetic single-domain Ni nanochevrons as “unchanged” permanently. That is, the switching of the single-domain state was permanent.

## 3. Fabrication

**Figure 3:**
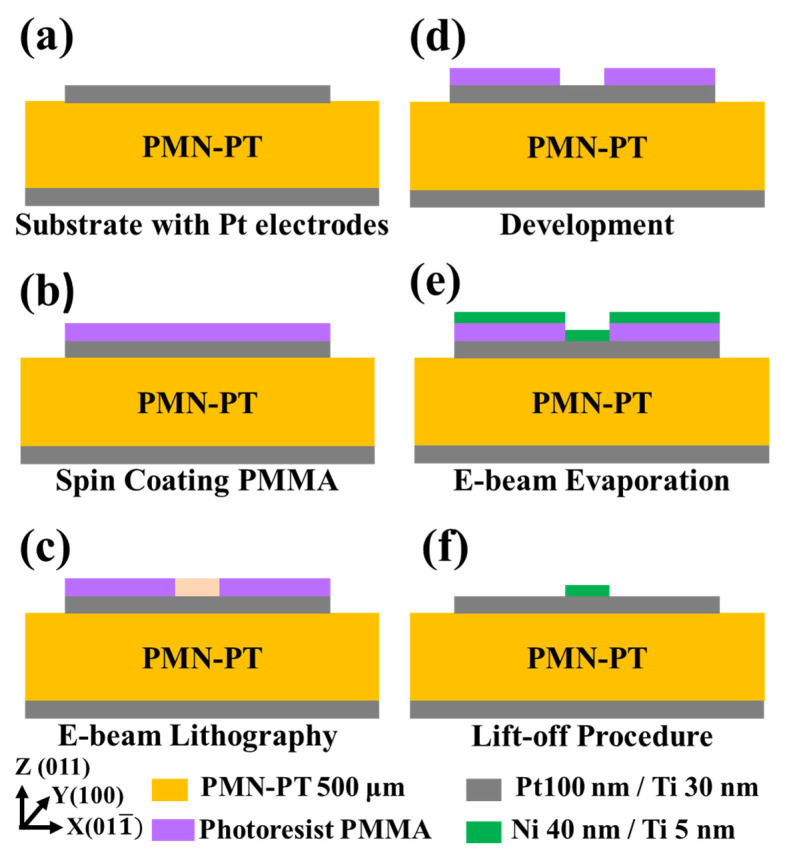
The fabrication process of the magnetic Ni nanochevron on the piezoelectric PMN–PT substrate: (**a**) Preparing a PMN-PT bulk as the substrate and then evaporating Pt/Ti Top and bottom electrodes on the substrate. (**b**) Spin coating of PMMA. (**c**) Patterning of the PMMA by e-beam writing. (**d**) Development of the Ni chevron template. (**e**) Deposition of the Ni/Ti layer by e-beam evaporation. (**f**) Patterning the Ni/Ti layer into a nanochevron using a lift-off process.

The fabrication process of the Ni nanochevron/PMN–PT layered structure is shown in Figure 3. Bulk piezoelectric PMN–PT was used as the substrate. E-beam evaporation was used to deposit Pt layers on the top and bottom sides of the PMN–PT layer (as top and bottom electrodes). The thickness of the Pt top electrode had to be within the range of 50 nm to 100 nm [4,12,13,16,17,18,22,27] to allow the strain from the piezoelectric PMN–PT layer below to be transmitted through it to reach the above magnetostrictive Ni nanochevron. After this, PMMA was spin-coated on the top surface of the Pt top electrode. Electron-beam writing was used to define/expose the chevron patterns in the PMMA layer. After the patterns were developed, e-beam evaporation was used to deposit Ni and Ti layers with thicknesses of 40 nm and 5 nm, respectively. After deposition, the lift-off process was performed to remove the undefined/unexposed parts of the PMMA layer with the unwanted parts of the Ni layer. This completed the fabrication of the Ni nanochevron/PMN–PT layered structure.

## 4. Results and Discussion

**Figure 4:**
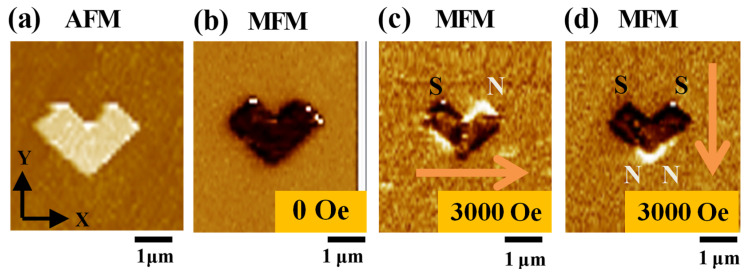
General geometric tomography and magnetic profile characterization results for the Ni nanochevron before performing our approach: (**a**) AFM image and (**b**) MFM image of Ni nanochevron captured immediately after fabrication (i.e., as-fabricated state). (**c**) MFM image captured after applying and then removing an x-axial magnetic field of 3000 Oe to initialize the single-domain state in the Ni nanochevron. (**d**) MFM image captured after applying and then removing a negative y-axial magnetic field of 3000 Oe to transform the single-domain state to a two-domain state. Orange arrows indicate the direction of the applied magnetic field. Labels N and S represent the magnetic north and south poles, respectively.

An AFM topographic image of a representative Ni nanochevron is shown in Figure 4a. The length, width, and thickness were approximately 1.83 um, 860 nm, and 45 nm, respectively. MFM magnetic domain images of the same Ni nanochevron are shown in Figure 4b–d. As fabricated, the Ni nanochevron exhibited an undistinguishable (chaotic) domain state. After applying and subsequently removing a positive x-axial magnetic field of 3000 Oe to the Ni nanochevron, a single-domain state was initialized in the Ni nanochevron (i.e., the initial state in our design), as shown in Figure 4c. After applying and then removing a negative y-axial magnetic field of 3000 Oe to the Ni nanochevron, its single-domain state was transformed into a two-domain state. In the two-domain state, the magnetic north poles were at the convex corner of the Ni nanochevron, whereas the magnetic south poles were at the ends of both sides of the Ni nanochevron. In this state, the magnetization of the Ni nanochevron followed the negative y-axial direction. This proves that by applying directional magnetic fields, we successfully transformed the magnetic domain pattern of the Ni nanochevron from a single-domain state to an unstable two-domain state, as expected.

**Figure 5:**
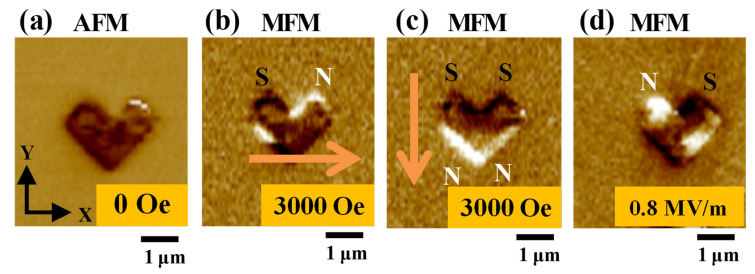
Results of magnetic-field-assisted electric-field-control magnetic single-domain transformation in a representative Ni nanochevron following our approach. MFM images of the Ni nanochevron: (**a**) after demagnetization; (**b**) after applying and then removing the 3000 Oe x-axial magnetic field, initializing a single-domain state; (**c**) after applying and then removing the 3000 Oe negative y-axial magnetic field, transforming the single-domain state into a two-domain state; and (**d**) after applying and then removing the 0.8 MV/m electric field, using the converse magnetoelectric effect to transform (release) the two-domain state back to one of two possible single-domain states. After the electric field was removed, the single-domain state remained stable. Thus, the domain pattern was switched. The orange arrow represents the direction of the applied axial magnetic field. Labels N and S represent the magnetic north and south poles, respectively.

After proving that the magnetic domain pattern of the Ni nanochevron could be transformed/controlled by applying magnetic fields, we subjected the Ni nanochevron to a standard demagnetization process to eliminate any residual effects of the previously applied 3000 Oe magnetic field, allowing the Ni nanochevron to return to its initial domain state. A typical result of Ni nanochevron demagnetization is shown in Figure 5a. Next, by repeating the above procedure, we again applied sequential magnetic fields to the Ni nanochevron to first reach the initial single-domain state (as shown in Figure 5b, similar to Figure 4c) and subsequently reach the two-domain state (as shown in Figure 5c, similar to Figure 4d). After this, we applied an electric field of 0.8 MV/m to the PMN–PT substrate (by applying a voltage across the top and bottom electrodes). Due to the converse magnetoelectric effect, the two-domain state was transformed to a single-domain state with the magnetization direction following the easy axis of the Ni nanochevron, as shown in Figure 5d. Hence, we successfully exploited the converse magnetoelectric effect to transform the two-domain state into a single-domain state. Furthermore, the single-domain state magnetization followed the negative x-axial direction. Comparing Figure 5d to Figure 5b shows that the north and south poles of the single-domain state were switched, demonstrating that the single-domain pattern was successfully switched (i.e., the magnetization direction of the single-domain state was successfully rotated by 180°).

In Figure 5b, two regions on the bottom side of the nanochevron seem to have north and south poles, indicating a multidomain pattern. This unexpected multidomain pattern sometimes appeared due to an unexpected domain pinning effect caused by unavoidable small, wavy, nanoscale geometric discontinuities (produced by e-beam writing, e-beam evaporation, and lift-off processes) along the edges and around the corners of the Ni nanochevron [13]. In general, as shown in Figure 5b, after magnetization by the magnetic field, the top-right and top-left ends of the Ni nanochevrons in the MFM images showed a north pole with a higher magnitude in a larger white region and a south pole with a higher magnitude in a larger dark region, respectively. However, this image shows not only these two stronger poles/regions at the two ends of the nanochevron but also the above-mentioned domain pinning effect near the bottom-left edge, indicating a multidomain pattern. Nevertheless, we focused on the domain state at the two ends of the nanochevron, showing stronger poles/magnitudes/regions (as expected by our design in Figure 2); thus, we did not dwell on the domain pattern at the bottom-left edge (which displayed smaller poles/magnitudes/regions and did not influence the two stronger poles/regions at the two ends of the nanochevron).

Although the above results show that the switched single-domain state conformed to one of the two expectations outlined in the design section (Figure 2c), the other expectation (i.e., another switched single-domain state) is not shown. According to our design, when we applied our approach to more magnetic single-domain Ni nanochevrons, roughly half of the resulting single-domain patterns would match one expectation, while the other half would match the other expectation. That is, the magnetization direction of roughly half of the single-domain Ni nanochevrons would be rotated by 180 degrees, while the other half would be returned to their original magnetization direction (no rotation). Thus, the probability for each case is close to 50%.

**Figure 6:**
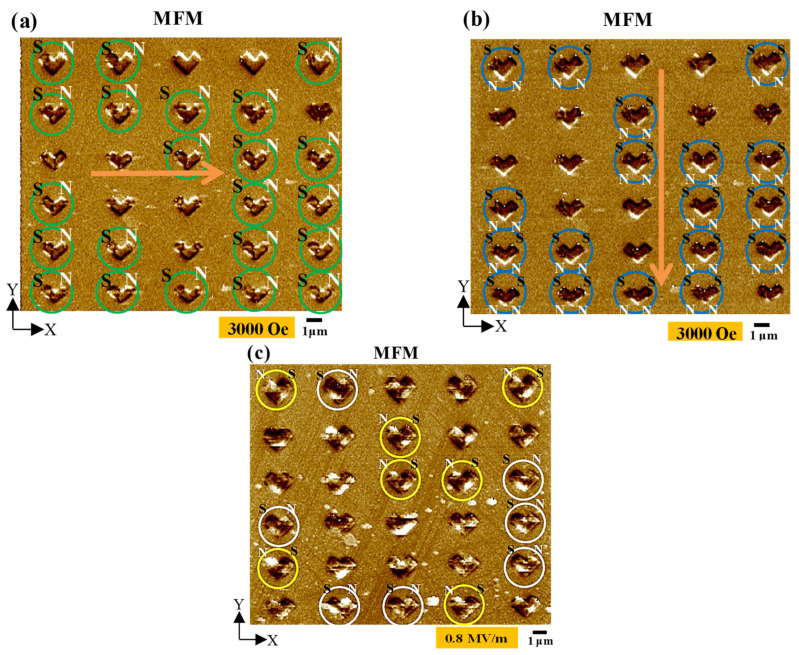
Results of magnetic-field-assisted electric-field-controlled magnetic single-domain transformation in the array of 30 Ni nanochevrons following our approach. MFM images of the Ni nanochevrons: (**a**) after applying and then removing the x-axial magnetic field of 3000 Oe, with 22/30 Ni nanochevrons initialized(magnetized) to a single-domain state (green circles); (**b**) after applying and then removing the negative y-axial magnetic field of 3000 Oe, with the single-domain state in 18 Ni nanochevrons (blue circles) among the previous 22 (green circles in (**a**)) transformed to the two-domain state; (**c**) after applying and then removing the electric field of 0.8 MV/m, producing the converse magnetoelectric effect. This transformed the two-domain state in 14 Ni nanochevrons (white and yellow circles) among the previous 18 (blue circles in (**b**)) back to one of two possible single-domain states. After the electric field was removed, the single-domain state in each Ni nanochevron remained stable. Thus, we achieved domain switching. The orange arrows represent the direction of the applied axial magnetic field. The labels N and S represent the magnetic north and south poles, respectively.

We fabricated an array of 30 magnetic single-domain Ni nanochevrons and repeated the above experiments using these Ni nanochevrons, providing a sufficient number of domain patterns to verify the above probability assumption. In the past, we presented initial, rudimentary nanochevrons with a very different length/width ratio to roughly demonstrate our idea at a conference. However, at that time, we only successfully fabricated four nanochevrons, and their domain patterns were more random than those presented herein due to the occurrence of a more serious domain pinning effect caused by the unrefined fabrication processes [33]. In addition, the four nanochevrons provided too few domain patterns to statistically verify the above assumption; thus, we required more nanochevrons with well-defined domain patterns. The experimental results for the array of 30 nanochevrons are shown in Figure 6. Figure 6a demonstrates that after applying the x-axial magnetic field of 3000 Oe to the array, most of the Ni nanochevrons were initially magnetized to display a single-domain state (indicated by green circles in the figure). The percentage of successfully initialized single-domain states across the whole array was approximately 73% (i.e., 22 out of 30 Ni nanochevrons). This means that most domains were magnetized along the x-axial direction, whereas the rest were magnetized in relatively random domain patterns (i.e., not a single-domain pattern along the x-axial direction). The unaligned condition of the latter domains could be attributed to the unavoidable domain pinning effect that sometimes occurs at the edges and corners, inside, and on the surface of nanochevrons. The domain pinning effect could be further attributed to any slightly wavy geometry along the edges, small geometric discontinuities around the corners, tiny particles inside, small dimples on the surface, or unstable conditions during the e-beam lithography or lift-off processes when fabricating the nanochevron. In the future, these issues can be avoided by using a higher-resolution e-beam writer to precisely fabricate the geometry of the nanochevron and a more stable e-beam evaporator to uniformly deposit the film.

After initialization, a negative y-axial magnetic field of 3000 Oe was applied to transform the single-domain state to a two-domain state, as shown in Figure 6b (the two-domain patterns are indicated by blue circles). The percentage of successfully transformed two-domain states among the whole array was approximately 82% (i.e., 18 out of 22 Ni nanochevrons). Finally, an electric field of 0.8 MV/m was applied to produce the converse magnetoelectric effect, thus transforming the two-domain state back to a single-domain state, as shown in Figure 6c. Figure 6c demonstrates that most of the two-domain states were transformed back to one of two possible single-domain states (indicated by white and yellow circles, respectively). The percentage of successfully transformed single-domain states was 64% (14 Ni nanochevrons in white and yellow circles out of the previous 22 in blue circles). Moreover, each of these single-domain nanochevrons (transformed from two-domain nanochevrons) exhibited one of two opposing magnetization directions, as expected. One magnetization direction followed the positive x-axis (indicated by the white circles in Figure 6c), while the other followed the negative x-axis (indicated by the yellow circles in Figure 6c).

To confirm the number of magnetic single-domain Ni nanochevrons that exhibited the desired results (i.e., the reversal of the initial magnetization direction), we compared the magnetization directions of the initialized/magnetized single-domain Ni nanochevrons (circled in green in Figure 6a) and the final transformed single-domain Ni nanochevrons (circled in white and yellow in Figure 6c). The comparison results showed that the magnetization directions of the single-domain Ni nanochevrons in the white circles were the same as those of the single-domain nanochevrons in the green circles. Furthermore, the magnetization directions of the single-domain nanochevrons in the yellow circles were opposite to those of the single-domain nanochevrons in the green circles. Most importantly, the numbers of single-domain nanochevrons circled in white and yellow were almost equal (as expected, according to our design). Additionally, the percentage of Ni nanochevrons exhibiting a transformation from the initial single-domain state to a multidomain state and, finally, a successfully switched single-domain state was 64% (i.e., 14 of the 22 initialized/magnetized Ni nanochevrons displayed domain switching). Thus, using our approach, the magnetization directions of approximately half of the single-domain nanochevrons were successfully switched (i.e., 180° magnetization rotation was achieved). The probability of successful switching demonstrated by our approach (approximately half) was as expected, according to our design.

In addition, to improve our work in the future, the number of unsuccessfully magnetized/transformed domains could be reduced by using more effective nanofabrication tools. These unsuccessfully magnetized/transformed domains sometimes occurred due to the unavoidable magnetic domain pinning effect frequently encountered in patterned nanostructures with complicated nanogeometries (nanochevrons in this work). The influence of domain pinning is more significant at locations with geometric discontinuities. Thus, the domains at the edges and corners of the nanochevrons were much more easily constrained by the domain pinning effect. If a nanochevron experienced a stronger domain pinning effect, its domain pattern would not be successfully magnetized or transformed, as in this study. Furthermore, geometric discontinuities were formed in the nanochevrons through two main fabrication processes, as described below. First, the e-beam writing process presented significant issues regarding the formation of geometric discontinuities. For nanopatterns with complicated nanogeometries, the e-beam writing process unavoidably formed a wavy side wall of nanopatterns in the PMMA layer. Consequently, after thin-film deposition, geometric discontinuities arose at the edges of the nanochevrons. Second, geometric discontinuities could also be formed at the edges of the nanochevrons during the lift-off process. That is, when the thin-film layer was removed (lifted off), it could have torn or peeled off the material at the edges of the nanochevrons. In the future, we will implement an improved e-beam writer with a higher resolution and a more thorough parameter optimization study for the lift-off process to reduce the occurrence of undesired geometric discontinuities in the nanochevrons. Thereby, the magnetic domain pinning effect will be mitigated, and the number of unsuccessfully magnetized/transformed domains decreased.

In conclusion, the results showed that our approach successfully and permanently switched the single-domain pattern of the Ni nanochevron/PMN–PT layered structure (rotating the magnetization direction by 180 degrees). In the future, our design can be improved by referencing representative simulation/modeling works in the same field to obtain more favorable results [34,35,36,37,38].

## 5. Conclusions

We demonstrated the magnetic-field-assisted electric-field-induced domain switching of a magnetic single domain in a multiferroic/magnetoelectric Ni nanochevron/PMN–PT layered structure. The experimental results showed that by sequentially applying a magnetic field of 3000 Oe along the axial direction (x-axial direction) and the transverse direction (y-axial direction) of a single-domain Ni nanochevron and finally applying an electric field of 0.8 MV/m to the layered structure, we successfully switched the single-domain pattern in the Ni nanochevron. After applying our approach, the single-domain patterns of approximately 50% of the Ni nanochevrons were permanently switched (i.e., the magnetization direction was permanently rotated by 180 degrees). This study represents an important advancement and design reference for developing future nanoscale electromagnetic systems.

## Data Availability

Data are contained within the article.
